# Temporal focusing multiphoton microscopy with optimized parallel multiline scanning for fast biotissue imaging

**DOI:** 10.1117/1.JBO.26.1.016501

**Published:** 2021-01-01

**Authors:** Chia-Yuan Chang, Chun-Yun Lin, Yvonne Y. Hu, Sheng-Feng Tsai, Feng-Chun Hsu, Shean-Jen Chen

**Affiliations:** a National Cheng Kung University, Department of Mechanical Engineering, Tainan, Taiwan; b National Chiao Tung University, College of Photonics, Tainan, Taiwan; c National Cheng Kung University, Department of Photonics, Tainan, Taiwan; d National Cheng Kung University, Department of Cell Biology and Anatomy, Tainan, Taiwan

**Keywords:** medical and biological imaging, fluorescence microscopy, nonlinear microscopy, three-dimensional microscopy

## Abstract

**Significance:** Line scanning-based temporal focusing multiphoton microscopy (TFMPM) has superior axial excitation confinement (AEC) compared to conventional widefield TFMPM, but the frame rate is limited due to the limitation of the single line-to-line scanning mechanism. The development of the multiline scanning-based TFMPM requires only eight multiline patterns for full-field uniform multiphoton excitation and it still maintains superior AEC.

**Aim:** The optimized parallel multiline scanning TFMPM is developed, and the performance is verified with theoretical simulation. The system provides a sharp AEC equivalent to the line scanning-based TFMPM, but fewer scans are required.

**Approach:** A digital micromirror device is integrated in the TFMPM system and generates the multiline pattern for excitation. Based on the result of single-line pattern with sharp AEC, we can further model the multiline pattern to find the best structure that has the highest duty cycle together with the best AEC performance.

**Results:** The AEC is experimentally improved to 1.7  μm from the 3.5  μm of conventional TFMPM. The adopted multiline pattern is akin to a pulse-width-modulation pattern with a spatial period of four times the diffraction-limited line width. In other words, ideally only four π/2 spatial phase-shift scans are required to form a full two-dimensional image with superior AEC instead of image-size-dependent line-to-line scanning.

**Conclusions:** We have demonstrated the developed parallel multiline scanning-based TFMPM has the multiline pattern for sharp AEC and the least scans required for full-field uniform excitation. In the experimental results, the temporal focusing-based multiphoton images of disordered biotissue of mouse skin with improved axial resolution due to the near-theoretical limit AEC are shown to clearly reduce background scattering.

## 1 Introduction

Multiphoton excited fluorescence (MPEF) microscopy is commonly adopted in academic studies, medical diagnoses, and industrial applications. The natural optical sectioning ability, due to the nonlinear phenomenon of multiphoton absorption via a high numerical aperture (NA) objective that focuses and generates a strong local electromagnetic field, could achieve micron/submicron spatial resolution and reconstruct three-dimensional (3D) fluorescence images for bio-structural analysis.^1^^–^^4^ The excitation wavelength of an ultrafast laser was chosen in the near-infrared region and low-absorption spectral window of a biological specimen so that MPEF microscopy could achieve a deep penetration depth in thick tissue. Furthermore, with the second-harmonic generation (SHG) signal that directly reveals highly polarizable molecules and non-centrosymmetric structures, the distribution of collagen and myosin could be imaged in the biospecimen without any staining needed.^5^^–^^7^ These advantages make MPEF and SHG suitable for *in vitro* and *in vivo* applications, such as imaging cortical vasculature or neuronal activities inside a mouse brain,^8^^–^^11^ high-resolution ophthalmoscope imaging for cellular retina structures,^12^ muscle disease measurement,^13^^–^^15^ and skin disease diagnosis.^16^ Based on the multiphoton excitation mechanism, temporal focusing can accommodate widefield and fast MPEF imaging due to the high-throughput illumination and detection ability.^17^^,^^18^ The diffraction element (e.g., a blazed grating) in temporal focusing multiphoton microscopy (TFMPM) is utilized for generating angular dispersion to separate different spectral components into different angles according to a diffraction equation. After passing through the 4-f imaging system, which consists of a collimating lens and objective lens, the spectral components overlap in phase to achieve temporal focusing on the focal plane of the objective. The broadened laser pulse is reconstructed to its original short pulse width to have sufficient peak power for widefield MPEF and SHG, and is less time-consuming than conventional point scanning microscopy.^19^^–^^22^ The axial excitation confinement (AEC) of TFMPM can achieve a few microns and depends on several system parameters, including the transform-limited pulse width, laser beam size, groove density of the diffraction grating, optical system magnification, and NA of the objective lens.^23^^–^^26^ With different modified system configurations, TFMPM is capable of various applications. For example, the fast 3D Brownian motion of beads can be directly tracked,^27^^–^^29^ and the neural activity of mouse can be analyzed with fast volumetric imaging.^30^^–^^33^ A heterodyne detection mechanism has been used for parallelized frequency-domain 3D fast fluorescence lifetime image measurement.^34^ TFMPM has also been successfully combined with optical tweezers for particle and cell trapping.^35^ Widefield multiphoton-induced ablation using high laser-pulse energy fluence in a TFMPM system has been demonstrated for chicken tendon machining, after which SHG imaging could be directly performed to view the machined tendon structure.^36^ Furthermore, an intensity mask can be integrated with the TFMPM for fast pattern machining and axially resolved microfabrication with various materials, including photoresist,^37^ bovine serum albumin,^38^ graphene oxide,^39^ and metal substrates.^40^ The desired intensity pattern can also be generated from a high-resolution phase mask applied by a liquid-crystal spatial-light modulator.^41^^–^^43^ A phase-contrast filter can be adopted to directly transform the phase mask into an intensity pattern to probe arbitrarily neural bodies or dendrites.^30^

To have superior contrast and high-quality images, various techniques have been developed for improving the spatial resolution of TFMPM and reducing the scattering effect on the excitation beam or emission signal. Widefield and axial-resolved TFMPM can be combined with photoactivated localization microscopy to investigate 3D cellular structures at super resolution.^44^ Nonlinear structured illumination microscopy (NSIM) expands the detection spatial frequency bandwidth of the objective to exceed the imaging resolution limit^45^ and can be applied to improve the TFMPM spatial resolution.^46^^–^^49^ The TFMPM image contrast can be further improved by eliminating the background noise. The HiLo technique^50^^–^^52^ and Hilbert transform^53^ can retrieve the in-focus image, which is modulated by the applied structural pattern, while rejecting the out-of-focus background. The optical pattern in the TFMPM changes the filling ratio on the back-focal plane of the objective and has been shown to affect the system AEC as well.^54^^,^^55^ With a more condensed AEC, improved contrast of the reconstructed in-focus images is expected. Adaptive optics (AO)^56^ could be further implemented for optical dispersion and aberration compensation to approach the system theoretical performance and excitation efficiency to enable deeper imaging inside biological specimens. A digital micromirror device (DMD) has the periodic structure of a micromirror array, and every micromirror can be either in the reflecting (ON) or blocking (OFF) state at a positive or negative tilt angle.^57^^,^^58^ If the laser beam incidents at the proper direction and angle, the DMD structure can function as a blazed grating providing angular dispersion that spatially separates the spectral components. Moreover, the ability to control every individual micromirror allows the light to be manipulated to form any desired optical pattern. With these DMD benefits, it is more compact and efficient to use a single DMD to replace these two key components of the diffraction element and active optical mask. The DMD-based TFMPM with patterned illumination has been successfully demonstrated for implementing the HiLo technique,^28^ NSIM,^47^ and Hilbert transform-based demodulation^53^ to improve the TFMPM image quality.

In addition to the widefield TFMPM, the line-scanning TFMPM features a configuration that includes a cylindrical lens that modifies the incident beam to a line shape, while a galvanometer mirror scans the laser line to form a full two-dimensional (2D) image. Accordingly, the line-scanning TFMPM has a superior AEC compared to widefield TFMPM and is equivalent to regular point-scanning MPEF microscopy since the back-aperture of the objective is filled and a full NA is utilized for focusing.^23^^,^^59^^,^^60^ The DMD has been shown to be capable of replacing both the cylindrical lens and galvanometer mirror to achieve a line-scanning mechanism that improves the AEC.^61^ The scattering effect, which limits the imaging depth in the specimen, can be suppressed by a virtual confocal setup achieved by carefully synchronizing the scanning galvanometer mirror with the rolling shutter of the sCMOS.^26^ Together with the acoustic optical modulator, the line-scanning TFMPM can also achieve structural illumination to enhance the image contrast and resolution.^62^ In this paper, a developed DMD-based TFMPM combined with the multiline scanning mechanism is demonstrated. With a single-line pattern applied by the DMD, the diffraction-limited line can achieve a 1.5  μm AEC, which is almost identical to the line-scanning-based TFMPM. To increase the frame rate for a full 2D image, a multiline pattern configuration, which is similar to pulse width modulation (PWM), is adopted. Although the frame rate becomes faster in tandem with the PWM period decrement, the periodic pattern structure induces the Talbot effect, which distorts the axial excitation intensity distribution and lowers the effective AEC. Both the simulation and experiment showed that the intensities of the subsidiary maxima were reduced to below 10% of the AEC’s central peak when the pattern period was over three-fold longer than the single-line width. With a PWM period four times the width of a single line, namely, a 25% duty cycle, we show that the multiline pattern can achieve an AEC of 1.7  μm, where the Talbot effect is eliminated, and the axial excitation maintains its best confinement as opposed to the 3.5  μm of the optimal conventional TFMPM. The beam shape on the back aperture is also shown to have a uniform distribution and high filling ratio, meaning that there is good utilization of the objective NA. A full 2D image can ideally be formed by scanning four patterns with spatial phase shift of π/2; furthermore, in practice, a uniform MPEF image can be obtained by eight patterns with spatial phase shift of π/4. All the AEC changes, together with different pattern structures, were experimentally measured and verified theoretical by simulation. With this multiline scanning-based TFMPM, we can have full 2D images with only a few pattern scans, instead of depending on the image pixels required when using conventional line scanning-based TFMPM. The DMD is used to dynamically update the multiline pattern with different spatial phases at its fastest binary pattern switching rate of 9.5 kHz. According to our system specifications, including DMD and optics, a multiline pattern with an AEC of 1.7  μm was verified as having the single-line width of 20 DMD-pixel and spatial period of 80 DMD-pixel. The MPEF images of biological specimens, such as mouse hair or adipose tissue in the subcutaneous layer in eosin-stained mouse skin, are demonstrated to show the improved image contrast due to the improved AEC and reduced scattering effect compared to conventional TFMPM.

## 2 System Setup and Simulation

### 2.1 Overall DMD-Based TFMPM System Setup

**Fig. 1:**
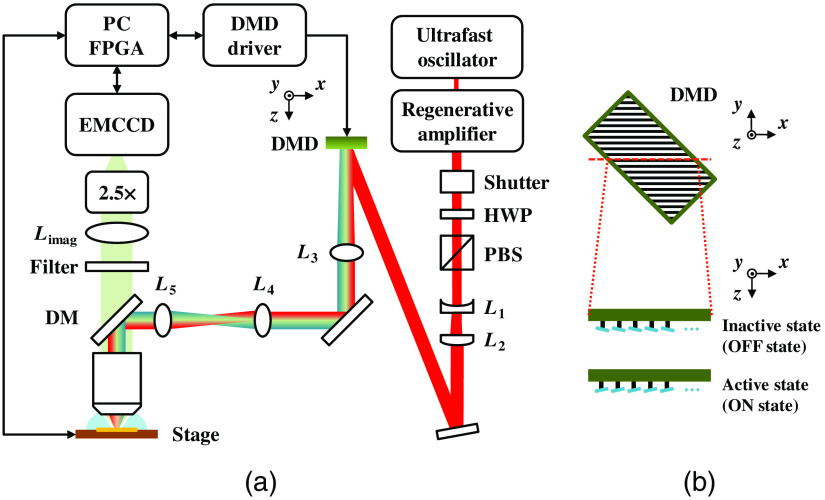
(a) Optical setup of the DMD-based TFMPM with parallel multiline scanning. (b) An illustration of DMD shows the line profile with the micromirrors tilted at −12  deg in an inactive (OFF) state, and the profile with micromirrors tilted at 12° in an active (ON) state.

The DMD-based TFMPM system is shown in Fig. 1. To have enough pulse energy to excite widefield or line-shaped MPEF signals, the Ti:Sapphire regenerative amplifier (Spitfire Pro XP, Spectra-Physics) seeded by a Ti:Sapphire ultrafast oscillator (Tsunami, Spectra-Physics) was adopted as the laser source and provides a maximum pulse energy of 400  μJ/pulse with a pulse width of 120 fs. The center wavelength is 800 nm and the repetition rate is 10 kHz. The fast mechanical shutter (VS14S-2-ZM-0-R3, Uniblitz) is open during the imaging process and can block the laser to avoid unnecessary exposure. After passing through the half-wave plate and the polarization beam splitter, it governs the laser output power and remains horizontal polarization. The DMD (DLP6500FYE, Texas Instruments) included in the evaluation module (DLP^®^ LightCrafter™ 6500, Texas Instruments) has a resolution of 1920×1080  pixels (1080 p) within a 0.65-in. chip. The pixel pitch is 7.56  μm and the mechanical tilt angle of every micromirror is ±12°. The binary pattern rate can be as high as 9.5 kHz with the DLPC900 digital controller. The image patterns were designed offline by MATLAB and uploaded to the internal memory of the controller to achieve the maximum pattern rate. Because the micromirror array structure has an orthogonal arrangement, and the hinge axis of the micromirrors runs along the diagonal of the chip, the DMD is rotated 45° to make the hinge axis parallel to y axis in Fig. 1. The DMD behaves like a brazed grating and the effective grating pitch is 10.69  μm with a blazed angle of 12°. The chosen sixth-order diffraction beam ensures that the overall efficiency is maximized according to the diffraction equation. Lenses $L_{1}$ (f=−75  mm) and $L_{2}$ (f=150  mm) expand the beam to make full use of the micromirrors by filling the DMD; meanwhile, $L_{3}$ (f=500  mm) and $L_{4}$ (f=200  mm) form a relay pair that extends the beam into the upright microscope (Axio Imager.A2m, Carl Zeiss, Germany). Lastly, $L_{5}$ (f=250  mm) and the water immersion objective (UPlanSApo 60XW/NA 1.2, Olympus, Japan) create the temporal focusing excitation. The optics were chosen to ensure that the beam shape on the back-focal plane of the objective nearly matches the diameter of the objective rear aperture for optimal AEC performance. The induced group velocity dispersion (GVD) from the system shifts the temporal focusing plane away from the conjugate plane of the generated pattern on the DMD. The resultant temporal focusing excitation and spatial focal plane mismatch would greatly lower the excitation efficiency and pattern contrast.^56^^,^^63^^–^^65^ In response, the built-in pulse compressor in the regenerative amplifier can be adjusted to compensate the overall system GVD and shift the temporal focusing excitation plane to match the spatial focal plane. Then, the generated optical pattern at the focal plane of the objective has superior excitation efficiency and could be imaged with high contrast. In this manner, the optics performs the optimal setup for temporal focusing excitation and patterned illumination. The MPEF and SHG pass through a dichroic mirror and optical filter and are detected by the electron-multiplying charge-coupled device (EMCCD) (iXon Ultra 897, Andor), which has 512×512  pixels and a pixel size of 16  μm. The 2.5× camera adaptor is used to magnify the excited fluorescence image to fill the EMCCD for optimal digital resolution. The single-pixel size of the EMCCD corresponds to 0.118  μm on the temporal focusing plane by calibration with the stage micrometer (#36-121, Edmund Optics Inc.) and is almost half of the theoretical diffraction limit of the system so that the digital resolution is sufficient according to the Nyquist criterion. By imaging the patterns on the DMD, the size of a single pixel on the DMD is calibrated and corresponds to 0.035  μm on the temporal focusing plane. The specimen is placed on the motorized stage (H101A ProScan, Prior Scientific) with a three-axis encoder to have precise position accuracy. The 3D images can then be captured by axially scanning with the fast piezo focusing stage (NanoScanZ 200, Prior Scientific) with a maximum traveling range of 200  μm. The PC includes a data acquisition card (PCIe-7842R, National Instruments) with a Virtex-5 LX50 field-programmable gate array that controls and communicates with all peripheral instruments and components.

### 2.2 Theoretical Simulation

**Fig. 2:**
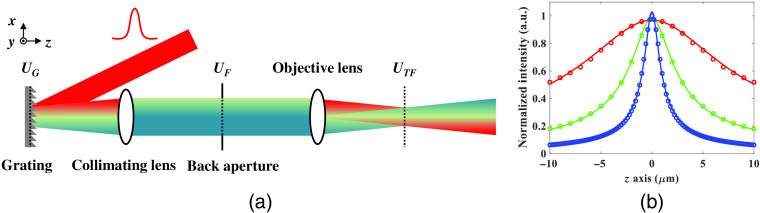
(a) Temporal focusing system setup. (b) Simulation results of axial intensity distribution with different laser spectral bandwidths. The red, green, and blue circles stand for bandwidth of 2, 4, and 8 nm. The solid lines are the fitted curves and the estimated FWHM are 22.8, 6.5, and 2.3  μm, respectively.

A conventional temporal focusing setup with a blazed grating, collimating lens, and an objective lens is shown in Fig. 2(a). The incident ultrashort laser pulse consists of different wavelengths that are interfered constructively in phase. The electric field $U_{G}$ at the grating plane is represented as a function of the superposition of different wavelengths with its initial phase according to the diffraction equation:

$$U_{G}(x_{G},y_{G};\omega )=\underset{\omega }{\sum }A_{G}(x_{G},y_{G};\omega )e^{-j\frac{2\pi c}{\omega }\;\sin\;\theta_{\omega }x_{G}},$$ (1)

where $A_{G}$ is the amplitude distribution of the corresponding frequency component and has a Gaussian distribution along the angular frequency ω. $\theta_{\omega }$ is the diffraction angle based on the grating equation and c is the speed of light in air. According to Fourier optics, the electric field $U_{F}$ on the Fourier plane equals the Fourier transform of $U_{G}$:^66^

$$U_{F}(x_{F},y_{F};\omega )=F_{\left(x_{G},y_{G})\to (x_{F},y_{F}\right)}\{U_{G}(x_{G},y_{G};\omega )\}.$$ (2)

It is noted that $U_{F}$ also represents the angular spectrum of $U_{G}$. On the other hand, it is assumed that the back-aperture acts like a spatial filter $L_{F}$ that passes only a limited bandwidth of angular spectrum of $U_{F}$. The final electrical field $U_{TF}$ on the temporal focusing plane could be represented as

$$U_{TF}(x_{TF},y_{TF};\omega )=F_{\left(x_{F},y_{F})\to (x_{TF},y_{TF}\right)}\{U_{F}(x_{F},y_{F};\omega )\cdot L_{F}(x_{F},y_{F})\}=\underset{\omega }{\sum }A_{TF}(x_{TF},y_{TF};\omega ).$$ (3)

As can be seen, $U_{TF}$ is obtained by summing all the frequency components. The two-photon excited fluorescence signal $I_{2p}$ is proportional to the square of the intensity profile $I_{TF}$ after Fourier transforming the frequency domain into the time domain on the temporal focusing plane:

$$I_{TF}(x_{TF},y_{TF};t)={\left|F_{\omega \to t}\{\underset{\omega }{\sum }A_{TF}(x_{TF},y_{TF};\omega )\}\right|}^{2}\;,$$ (4)

$$I_{2p}(x_{TF},y_{TF};t)\propto {\left|I_{TF}(x_{TF},y_{TF};t)\right|}^{2}.$$ (5)

To obtain the overall temporal focusing intensity distribution along the z direction, we start with the angular spectrum propagation. Based on Helmholtz equation, the effect of the axial propagation of the angular spectrum is the product of the relative phase term and its corresponding component.^66^ Furthermore, the corresponding angular spectrum of the electric field on the temporal focusing plane is the electric field on the Fourier plane, so we can simply apply the propagation phase term before we perform the Fourier transform for $U_{TF}$ ($x_{TF}$, $y_{TF}$, Δz; ω). Finally, the $I_{TF}$ ($x_{TF}$, $y_{TF}$, Δz; t) can be calculated:

$$I_{TF}(x_{TF},y_{TF},\Delta z;t)={\left|F_{\omega \to t}\{\underset{\omega }{\sum }A_{TF}(x_{TF},y_{TF},\Delta z;\omega )\}\right|}^{2}\;={\left|F_{\omega \to t}\{\underset{\omega }{\sum }F\{A_{F}(x_{F},y_{F},\Delta z;\omega )\cdot L_{F}(x_{F},y_{F})\}\}\right|}^{2}\;={\left|F_{\omega \to t}\{\underset{\omega }{\sum }F\{A_{F}(x_{F},y_{F};\omega )\cdot L_{F}(x_{F},y_{F})\cdot e^{jk_{z}(\frac{x_{F}c}{\omega f},\frac{y_{F}c}{\omega f})\Delta z}\}\}\right|}^{2},$$ (6)

where $k_{z}$ is the z component of the wave number and f is the focal length of the objective. We can directly obtain $I_{TF}$ at different z position based on Eq. (6) and the overall lateral and axial $I_{2P}$ distribution can be fully simulated. The AEC can be calculated by fitting the axial $I_{2P}$ data points with the model of the two-photon excitation response of the temporal focusing system.^23^^,^^63^ The AEC is defined as the full width at half maximum (FWHM) of the fitted curve. Based on theoretical model $I_{TF}$, Fig. 2(b) shows the simulation results of axial intensity distribution with different laser spectral bandwidths. The circles are the simulation data, and the solid lines are the fitted curves. The color of red, green, and blue stand for bandwidth of 2, 4, and 8 nm, and the fitted AEC are 22.8, 6.5, and 2.3  μm, respectively.

### 2.3 System Performance

**Fig. 3:**
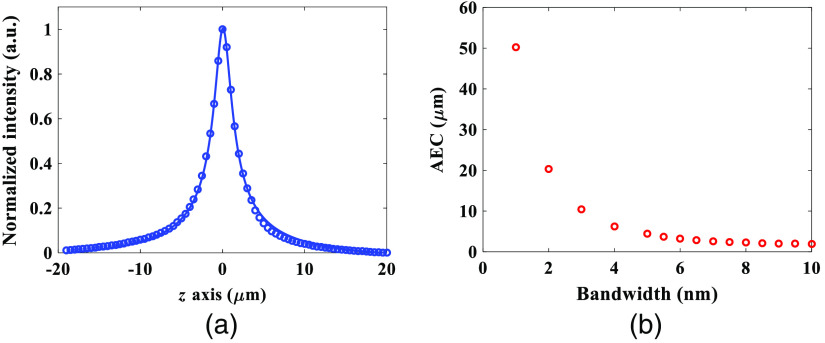
(a) AEC of the DMD-based TFMPM. Experimental measurement (blue circles) and fitted curve (blue line). The estimated FWHM is 3.5  μm. (b) Simulation result of the AECs of the TFMPM with different laser spectral bandwidths.

The AEC of the DMD-based TFMPM shown in Fig. 1 is estimated by experimentally measuring the axial intensity profile of a thin film doped with Rhodamine 6G (R6G) dye (<200  nm thickness). Figure 3(a) shows the data points of the average fluorescence intensity at every z step of 0.5  μm (blue circles). According to the fitted curve (blue line) in Fig. 3(a), the AEC of the system is estimated to be 3.5  μm. To find the effective laser spectral bandwidth, the system in Fig. 1 is converted into the conventional temporal focusing system setup as shown in Fig. 2 and performs the simulation based on the theoretical model described in Sec. 2.2. The sixth-order diffraction beam of the DMD used in TFMPM effectively equals to the first-order diffraction beam of a 561  grooves/mm blazed grating. The relay pair $L_{3}$ and $L_{4}$ together with $L_{5}$ match the collimating lens of 625 mm in Fig. 2; meanwhile, the 60× Olympus objective lens has an effective focal length of 3 mm. With Eq. (6), we can simulate the axial intensity change for every Δz of 0.2  μm in one direction from the temporal focusing plane (z=0) with the assumption that the curve is symmetric in order to reduce the overall computation time for simulation in MATLAB. With different laser spectral bandwidth value, the AEC is calculated from the curve that is fitted to the simulated axial intensity data points in each case. The simulation result of the AEC of the TFMPM varies with respect to the laser spectral bandwidth is shown in Fig. 3(b), and the effective full spectral bandwidth of laser is estimated to be 5.7 nm according to the experimental measurement of the 3.5  μm AEC. With this estimated effective spectral bandwidth value, we can discuss how the system AEC would be influenced by the generated pattern on the DMD and the limited size of the back-aperture of the objective, $L_{F}$. Furthermore, the DMD pattern is able to be well-designed to improve the AEC and verified by simulation.

## 3 Experimental Results

### 3.1 Line Scanning Performance with Line Pattern of Different Line Width

**Fig. 4:**
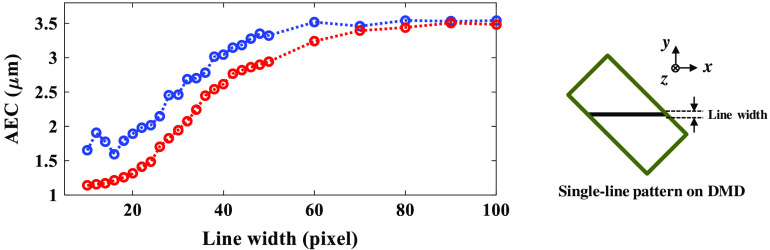
Curve of the AEC changes with different line width patterns on the DMD. The blue and red circles are experimental measurements and simulation data, respectively.

The line-scanning TFMPM has a superior AEC theoretically because of the full utilization of the NA of the objective. With the DMD-based TFMPM, we can generate line patterns on the DMD to achieve a line-scanning mechanism. The line was chosen parallel to the blazed direction. Although synchronizing the DMD with the PC via the video interface is more convenient and intuitive, serious background noise due to the micromirrors switching noise^67^ will arise, which in turn limits the pattern switching rate. Instead of using the video mode, the patterns are first uploaded into the internal memory of the DMD controller and displayed on the DMD in sequence when triggered so that the background micromirror switching noise could be eliminated. Patterns with different line widths are applied and the AEC curves are experimentally acquired by axially scanning the R6G thin film. The fitted AEC values with their corresponding line widths are plotted in Fig. 4 as blue circles. When increasing the line width, the AEC will converge to the value of 3.5  μm, which is reasonable since the line pattern is so thick that it acts as the widefield TFMPM. On the other hand, reducing the line width can push the AEC to nearly 1.5  μm, which is equivalent to line-scanning TFMPM. The trend for how the AEC changes according to the influence of the pattern $P_{\mathrm{DMD}}$ is validated by simulation described in detail in Sec. 2.2:

$$U_{F}^{\prime }(x_{F},y_{F})=F\{U_{G}(x,y_{G})\cdot P_{\mathrm{DMD}}(x_{G},y_{G})\},$$ (7)

$$U_{TF}^{\prime }(x_{F},y_{F})=F\{U_{F}^{\prime }(x_{F},y_{F})\cdot L_{F}(x_{F},y_{F})\}.$$ (8)

The laser parameter of the effective full spectral bandwidth is estimated by the measured AEC value according to the simulated relationship between the AEC and spectral bandwidth in Fig. 3. The ${U^{\prime }}_{F}$ on the Fourier plane is the electric field that is the Fourier transform of the product of $U_{G}$ and the applied pattern $P_{\mathrm{DMD}}$ on the DMD. The ${U^{\prime }}_{TF}$ on the temporal focusing plane is the Fourier transform of the ${U^{\prime }}_{F}$ after the spatial low-pass filter due to the limited back-aperture size of the objective. The theoretical AEC profile can be performed according to Eq. (6) with ${U^{\prime }}_{TF}$. The simulated AECs with respect to different $P_{\mathrm{DMD}}$ of different line widths are shown in Fig. 4 and plotted as red circles. The simulation matches the experimental data and confirms the correctness of the simulation method. According to Fig. 4, the line pattern with fewer than 20 pixels does not have much AEC improvement and has low reflection efficiency for excitation due to fewer active micromirrors on the DMD. For the single-line scanning, we take 20 pixels to be the minimum width of a single line before the AEC starts to grow. In addition, the main central peak of the diffraction limit focus for the system shown in Fig. 1 at an excitation wavelength of 800 nm based on the Rayleigh criterion is 0.81  μm which corresponds to around 23 DMD pixels, so a line pattern has a line width <20  pixels on DMD would be meaningless.

### 3.2 Parallel Multiline Scanning

**Fig. 5:**
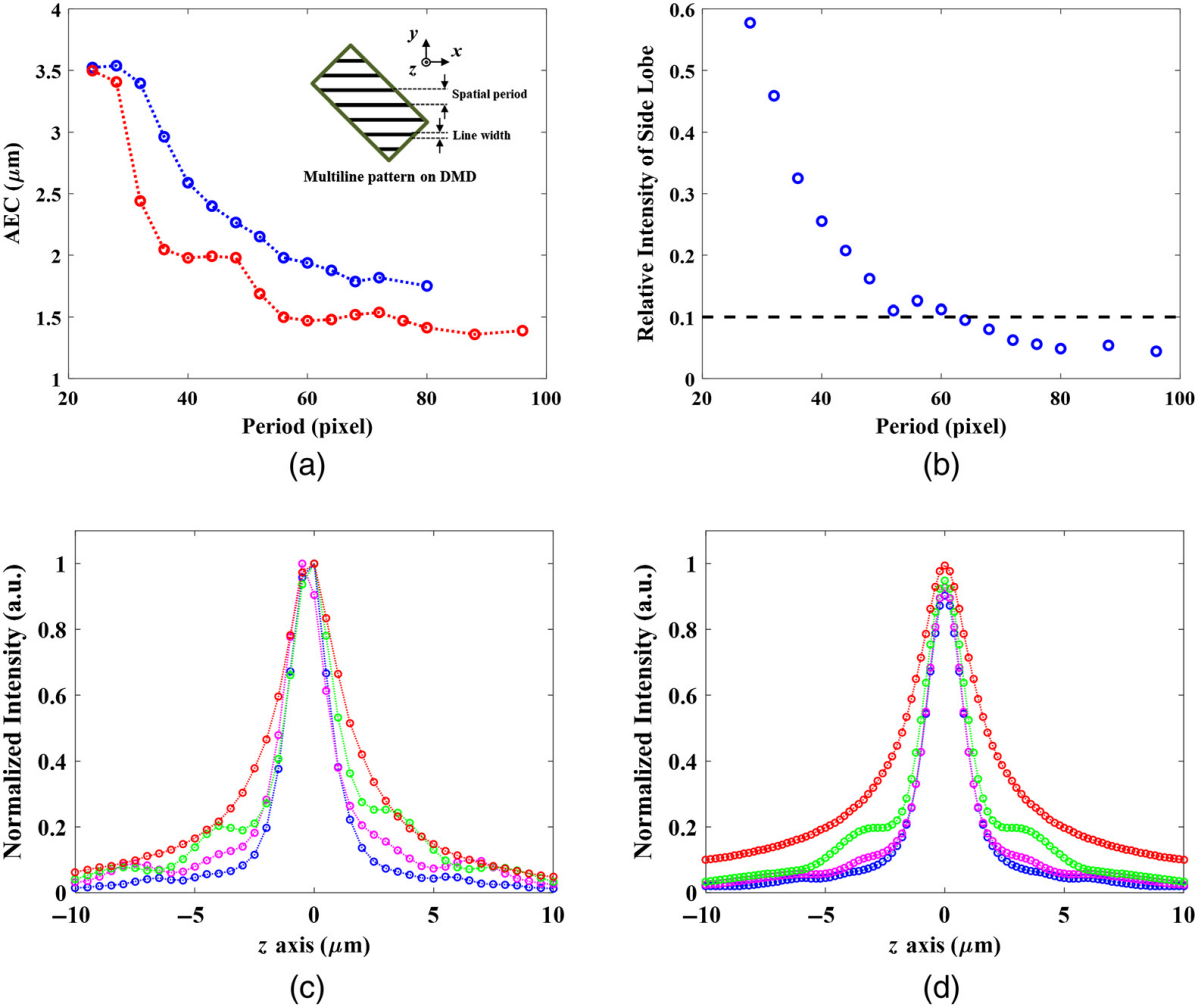
(a) The AEC curve changes with the pattern of the parallel 20-pixel lines with different periods on the DMD. The blue circles are the experimental measurement, while the red circles are the simulation data. (b) The Talbot effect-induced side lobe intensity relative to the main lobe intensity with respect to different pattern periods. The blue circles denote the simulation data and the black dashed line indicates 0.1; axial intensity curves with different period patterns: (c) experimental measurement and (d) simulation. The red, green, magenta, and blue circles stand for 24-, 44-, 60-, and 80-pixel period patterns, respectively. The side-lobes induced by the Talbot effect are clearly observed both in the experiment and simulation. The side-lobes move outwards, but the effect on the AEC is minimized when the pattern period is increased.

**Fig. 6:**
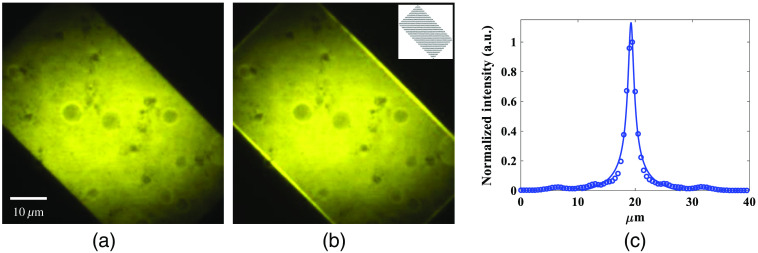
Widefield two-photon excited fluorescence image of an R6G thin film with the DMD-based TFMPM with (a) all DMD pixels active and (b) parallel multiline scanning. The bright signal on edges are due to the fixed margin of DMD which would keep reflecting the laser for excitation during scanning. (c) AEC of (b), with the experimental measurement (blue circles) and fitted curve (blue line). The FWHM is estimated as 1.7  μm.

Conventional line-scanning TFMPM requires scanning a line to form a widefield image. If the image has 512×512  pixels, it takes 512 scans to form the full image. The DMD-based TFMPM has the ability to generate multiline patterns and is capable of scanning the patterns to render an image. We have verified that a 20-pixel-width line can have an AEC of 1.7  μm, which approaches the theoretical limit according to the simulation. Next, we experimentally measure the AEC with the multiline pattern which has the single-line width of 20 pixels and different spatial periods. In Fig. 5(a), the blue circles show the experimental data while red circles indicate the simulation results. When the spatial period is shorter, the pattern becomes similar to the pattern of all pixels are in the “ON” state and its AEC approaches the performance of conventional TFMPM. On the other side, together with the reduction of the pattern period, the subsidiary maxima of AEC induced by the Talbot effect move toward to the main peak and affect the resultant AEC. In the simulation, the relative intensities of the subsidiary maxima of the Talbot effect decrease together with the increased pattern period as shown in Fig. 5(b). The resultant AEC is improved when the Talbot effect is ceased. In the experiment, Figure 5(c) shows the measured axial intensity curve with different spatial periods of the pattern. The red, green, magenta, and blue circles stand for 24-, 44-, 60-, and 80-pixel periods, respectively. The simulated data of the same pattern periods as Fig. 5(c) are shown in Fig. 5(d). The overall AEC changes, together with the pattern period and the subsidiary peaks status trend, can all be verified with the theoretical simulation. The Talbot effect on the AEC can be minimized when the subsidiary peaks are moved away from the main peak and the relative intensity of subsidiary peaks to the main central peak reduces to below 10% in Fig. 5(b). In other words, the widened AEC due to the subsidiary peaks can be relieved when the pattern period is larger than three times the width of a single line. In practice, four times the width of a single line would result in the best AEC, while longer period patterns seem not to provide much AEC improvement and more patterns are required to form a complete image. Ideally, since the pattern has 25% duty cycle, four patterns with different spatial phases (0, π/2, π, and 3π/2) should be sufficient to form a widefield image with the improved AEC. However, the limited spatial bandwidth of the optical system would result in a periodic background on the final image when these four multiline patterns are applied. In practice, the eight multiline patterns with π/4 phase increment, $P_{n}$ and n=0,1,…,7, are designed for scanning to ensure a uniform excitation image:

$$P_{0}(y_{G})=\{\begin{array}{ll} 1, & 0\leq mod(y_{G},80d_{\text{pitch}})<20d_{\text{pitch}}\\ 0, & 20d_{\text{pitch}}\leq mod(y_{G},80d_{\text{pitch}})<80d_{\text{pitch}} \end{array}\;P_{n}(y_{G})=P_{0}(y_{G}-10nd_{\text{pitch}}),\;n=1,2,\ldots ,$$ (9)

where $d_{\text{pitch}}$ is the size of the DMD pixel pitch. A value of 1 indicates an active state of the responding DMD pixel and 0 indicates an inactive state. With the DMD-based TFMPM, when all DMD pixels are active, it acts the same as a conventional TFMPM. The corresponding widefield two-photon excited fluorescence image of the R6G thin film is shown in Fig. 6(a). On the other hand, the eight multiline patterns described in Eq. (9) are continuously scanned at 9.5 kHz during the EMCCD exposure time. The acquired image is shown in Fig. 6(b). There is no obvious stripes and artificial shadows in the image, which shows good excitation uniformity as the same as the original widefield image. The bright signals on the edges of the image in Fig. 6(b) is due to the fixed margin of DMD. The margin always maintains the laser to excite the sample while the multiline pattern is scanning during the EMCCD exposure. The insets of Fig. 6(b) show one of the multiline patterns described in Eq. (9). The AEC of the parallel multiline scanning-based TFMPM is verified as 1.7  μm according to the fitted blue curve in Fig. 6(c). The blue circles indicate the experimental average intensity data at different axial depths, and the observable small side peaks of the blue circles are quite small compared to the main peak. Therefore, they will not result in any background noise or ghost signals in the acquired widefield TFMPM images.

**Fig. 7:**
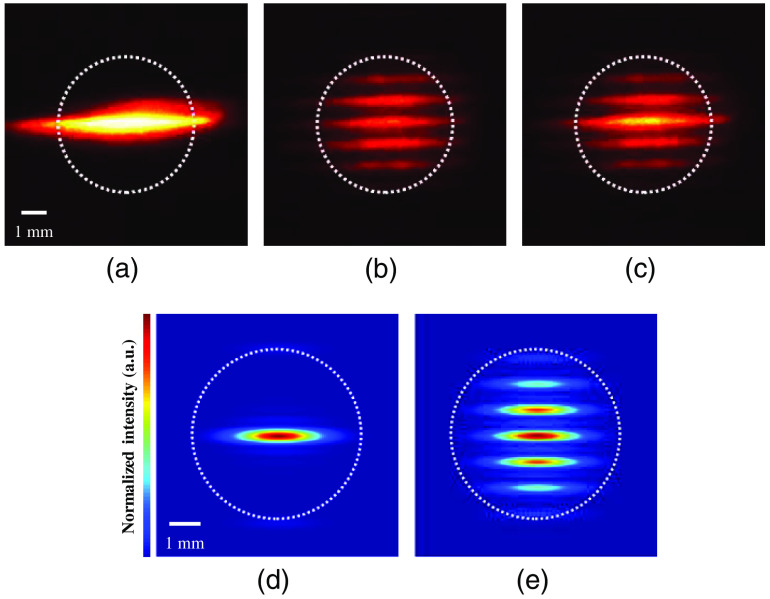
The beam shape on the back aperture of the objective with: (a) all DMD pixels active and captured by digital camera; (b) in-process parallel multiline pattern scanning; (c) multiline pattern sequences include a uniform pattern with all DMD pixels active (ON state); (d) all DMD pixels active in the simulation; and (e) parallel multiline pattern scanning in simulation. The white dotted circle indicates the boundary of the objective aperture.

Figure 7(a) shows the back-aperture image of the conventional TFMPM. The line-shaped beam captured with the digital camera (COOLPIX P7000, Nikon, Japan) is horizontally expanded due to the angular dispersion induced by the periodic structure of the DMD. The simulation described in Sec. 2.2 yields a similar beam shape as shown in Fig. 7(d). The white-dashed circle indicates the boundary of the objective back aperture. It was noted that the low filling ratio of the incident beam on the back-aperture plane deteriorates the effective objective NA and lowers the system AEC. On the other hand, the parallel multiline-scanning mechanism means that every multiline pattern on DMD induces its spatial-modulated shape in addition to the dispersion line-shape, both of which result in a diffraction pattern that has a higher filling ratio and a more uniform beam shape on the back-focal aperture of the objective. In this manner, both a higher utilization of effective objective NA and superior AEC are realized. Since all the multiline patterns described in Eq. (9) have the same line width and period except the phase term, the resulting beam shapes remain the same on the back-aperture plane and the multiline patterns are continuously switched for parallel multiline scanning. An experimental back-aperture image captured during scanning and a simulated image are shown in Figs. 7(b) and 7(e), respectively. On the other side, if the scanning multiline pattern sequences include any unwanted pattern, e.g., uniform pattern with all DMD pixels active when the video mode of the DMD controller is adopted, the beam shape on the back-focal aperture of the objective is shown in Fig. 7(c). The beam shape is less uniform than Fig. 7(b) and more intense in central line. The non-uniform beam shape will result wider AEC and deteriorate the system performance.

### 3.3 Biotissue Imaging with Parallel Multiline Scanning-Based TFMPM

**Fig. 8:**
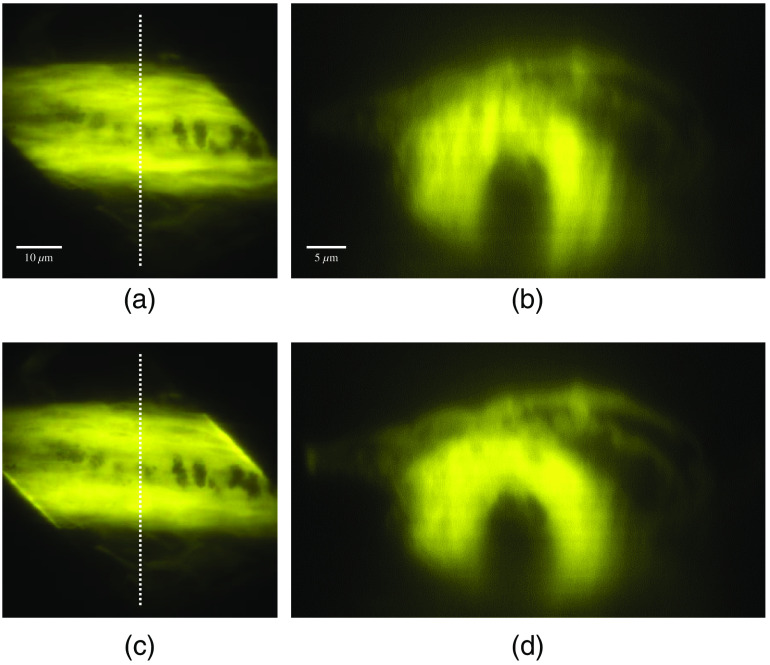
Widefield two-photon excited fluorescence images of mouse hair in eosin-stained skin with a conventional TFMPM on (a) the xy plane and (b) the yz plane, and with the parallel multiline pattern scanning-based TFMPM in (c) the xy plane and (d) the yz plane.

**Fig. 9:**
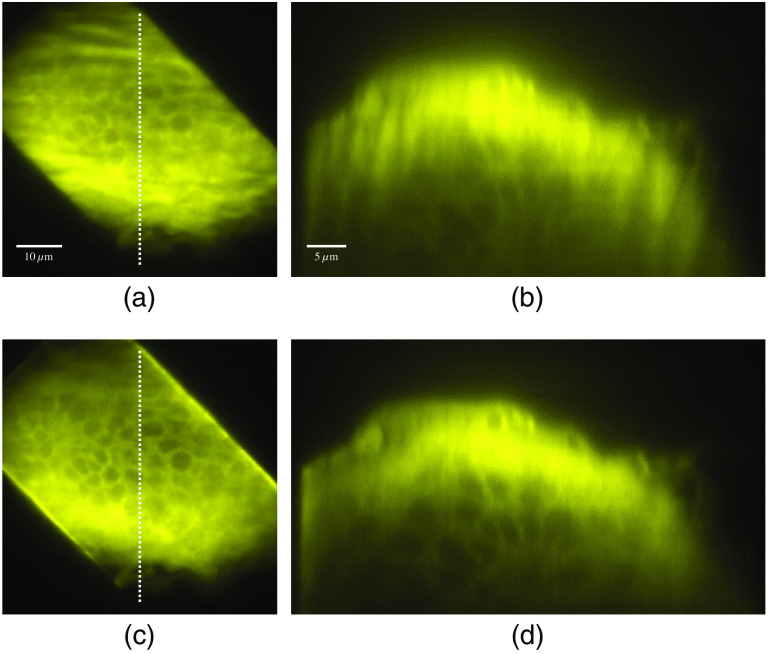
Widefield two-photon excited fluorescence images of adipose tissue in the subcutaneous layer of eosin-stained mouse skin with a conventional TFMPM on (a) the xy plane and (b) the yz plane, and with the parallel multiline pattern scanning-based TFMPM in (c) the xy plane and (d) the yz plane. A comparison between these two different imaging methods at different z-stacks is given in Video 1 (mp4, 1.35 MB) https://doi.org/10.1117/1.JBO.26.1.016501.1, with both different sectional images shown in Video 2 (mp4, 1.0 MB) https://doi.org/10.1117/1.JBO.26.1.016501.2.

The developed parallel multiline scanning-based TFMPM system was demonstrated to deliver an improved AEC of 1.7  μm. With the enhanced AEC, the axial excitation region of the specimen is better confined, and less background noise will be induced from the out-of-focus region. With the improved axial-resolving ability, the image contrast is obviously superior to the original TFMPM image. The complex biotissue of an eosin-stained mouse skin specimen is used for demonstration. Figure 8(a) shows the mouse hair in dermis, and the medulla structural pattern can be clearly observed. The sectional image on the yz plane of the white-dashed line is shown in Fig. 8(b). The image blur is resolved with the parallel multiline scanning technique that improved the AEC. The images are shown in Figs. 8(c) and 8(d). The details can be clearly observed in the adjacent dermis and in the sectional images of Fig. 8(d) as well. The adipose tissue in the subcutaneous layer imaged with a conventional TFMPM on the xy plane and the yz plane indicated by the white-dashed line are shown in Figs. 9(a) and 9(b), respectively. In comparison, a fluorescence image of the xy plane and sectional yz plane produced with the AEC-improved and parallel multiline scanning-based TFMPM is shown in Figs. 9(c) and 9(d), respectively. The structural shadow that appears above the image in Fig. 9(a) is attributed to the original broad AEC and scattering. With the parallel multiline scanning, Fig. 9(c) shows an image with improved contrast and effectively eliminated background disturbance. Moreover, the reconstructed images on the sectional yz plane display improved contrast and more observable details. The power efficiency of the DMD-based TFMPM is around 20% when all DMD pixels are active. To avoid the damage to DMD by the regenerative amplifier with long exposure time, the laser power to DMD is limited to 70 mW, which corresponds to 14 mW on specimen. With the multiline pattern with 25% duty cycle for the parallel multiline scanning, the actual power on the specimen is expected to be around 3.5 mW. The limited power excitation only allows the system to excite and receive the fluorescence signal at the depth of 60  μm in this complex eosin-stained mouse skin specimen. However, the contrast improvement of the reconstructed image is clearly observed in the limited depth.

## 4 Conclusions

TFMPM has the ability for widefield and axially resolved MPEF images with an AEC of a few microns and is capable of direct observation of fast 3D Brownian motion, neural dynamics analysis, and various other applications. However, the low effective-objective NA utilization due to the low filling ratio of the incident beam on the back aperture of the objective results in a limited AEC. The line-shaped beam on the back-aperture plane is horizontally expanded by the angular dispersion induced by the diffractive element in the system. It is impossible to further improve the AEC by simply optimizing the optics in the system. On the other hand, line-scanning TFMPM uses a line-shaped laser beam focused by a cylindrical lens with continuous beam scanning by mechanical scanner to form a 2D image. The beam can be vertically expanded on the back aperture of the objective so that it almost fills the aperture area and fully utilizes the NA to achieve an optimal AEC. Although the AEC is improved, the total acquisition time for forming a complete 2D image strongly depends on the numbers of scanning line. As such, the numerous scans required for 2D imaging might restrict the applications.

In this paper, the developed parallel multiline scanning-based TFMPM shows that the multiline pattern with a duty cycle of 25% yields an AEC close to that of a line scanning-based TFMPM, and was verified with theoretic simulation. With this approach, only eight multiline patterns are needed for uniform excitation and imaging without the dependence of the image pixel-number as single-line scanning-based TFMPM required. The DMD provides pattern with high spatial resolution due to the dense pixels and is utilized for the switch between conventional TFMPM (all DMD pixels are active), line scanning-based TFMPM (single-line width <20  pixels are active), and the multiline scanning-based TFMPM configuration. So that we can do fair and precise comparison and performance verification in the same optical setup although there are some limitation factors of DMD including the limited DMD aperture size and low diffraction efficiency. The scanning multiline pattern is able to be generated and applied on the blazed grating with higher diffraction efficiency and integrated with faster scanning mechanism (like resonant scanner or polygon scanner) and multi-beam configuration to realize the parallel multiline scanning mechanism. We have proved the optimized multiline pattern is the pattern with the active diffraction-limited line width and duty cycle of 25%, and it requires only eight scans for uniform excitation while maintaining the best AEC. With this configuration, the parallel multiline scanning-based TFMPM has an improved AEC of 1.7  μm compared to the 3.5  μm AEC of a conventional TFMPM and the Talbot effect induced subsidiary peaks on the AEC is reduced below 10%. A biotissue specimen was also imaged to demonstrate the image-quality improvement with the superior AEC. The background noise was eliminated and additional details could be observed on both the xy plane and reconstructed yz sections of both the mouse hair and adipose tissue.
